# David and Goliath: chemical perturbation of eukaryotes by bacteria

**DOI:** 10.1007/s10295-015-1686-6

**Published:** 2015-10-03

**Authors:** Louis K. Ho, Justin R. Nodwell

**Affiliations:** Department of Biochemistry, University of Toronto, 1 King’s College Circle, Toronto, ON M5S 1A8 Canada

**Keywords:** Actinomycetes, Eukaryotes, Target diversity

## Abstract

Environmental microbes produce biologically active small molecules that have been mined extensively as antibiotics and a smaller number of drugs that act on eukaryotic cells. It is known that there are additional bioactives to be discovered from this source. While the discovery of new antibiotics is challenged by the frequent discovery of known compounds, we contend that the eukaryote-active compounds may be less saturated. Indeed, despite there being far fewer eukaryotic-active natural products these molecules interact with a far richer diversity of molecular and cellular targets.

## Introduction

Actinobacteria are gram-positive bacteria that are ubiquitous in soil and marine sediments. There are an exceptionally diverse number of genera that include *Streptomyces, Micromonospora*, *Amycalotopsis, Salinospora, Saccharopolyspora, Actinomycetes* and many others. These organisms have evolved striking developmental and physiological adaptations that allow them to compete and survive in crowded environments. One notable feature is their ability to produce biologically active small molecules, referred to variously as ‘natural products’, ‘secondary metabolites’, and ‘specialized metabolites’ that have been isolated and used as antibiotics and other therapeutics.

Drug discovery based on mining metabolites from actinobacteria, based on enormous screens of culture supernatants against pathogenic bacteria, was successful from 1950 to 1970 and generated many of the antibacterial drugs we now have at our disposal. However, the repeated re-discovery of known antibacterials from this source led to the abandonment of this approach during the 1990s. The prevailing view by the mid-1990s was that there was no new chemical diversity left to discover from this source. However, the advent of genome sequencing revealed that the reservoir of biosynthetic genes for these compounds, including polyketides, non-ribosomal peptides and other classes, is much larger than had been previously appreciated [19, 90, 138]. We now know that each actinomycete genome encodes 20–50 biosynthetic gene clusters for secondary metabolites [134]. It is not currently possible to assign a product structure or biological activity to most of these biosynthetic pathways. Indeed, many of the secondary metabolites produced by well-characterized model strains such as *Streptomyces coelicolor, Streptomyces griseus* and *Streptomyces avermitilis* are still unknown. As a result, there has been renewed emphasis on the discovery and characterization of these cryptic metabolites through the use of new bioinformatic approaches, innovative culture techniques, genetic manipulation, chemical manipulation and new screening regimens [40, 41, 69, 194, 103, 109, 117, 127, 136, 151, 165, 173].

There are several explanations for why so many secondary metabolites have eluded discovery. One view is that many secondary metabolic genes are expressed at low levels in the laboratory and that their products cannot therefore be easily detected. Another is that there may be a ‘screening bias’ in the existing discovery regimens. For example, the vast majority of screening has been for antibiotics—it is possible that some of the uncharacterized chemical matter act on other targets.

The primary focus in this field since its inception has been on the discovery of new antibiotics. This charge has been renewed most recently due to a pressing need for new approaches to treating resistant pathogens [15, 16]. Nevertheless, we wonder whether some of the diversity of natural products is being overlooked. It is known for example, that there are many secondary metabolites that interact with eukaryotic cells (Table 1). These include known secondary metabolites commonly used as clinical antifungal, anticancer, immunosuppressive, antiangiogenic, and antiprotozoal drugs [20]. As we will describe in this review, the target diversity of these eukaryote-directed compounds exceeds that of the antibacterials. Indeed, another explanation for the failure to discover some of this diversity could be that the screening bias towards finding antibacterials has caused compounds that are expressed in the lab to go undetected due to the fact that the wrong assay was employed. Our intent, therefore, is to examine a selection of known eukaryote-directed secondary metabolites in the interest of stimulating the discovery of secondary metabolites that act on eukaryotic targets. In addition to providing new probes of intricate biological pathways, such molecules could provide leads for new therapeutics against many diseases.

**Table 1:** The eukaryotic targets of actinomycete metabolites

Drug	Producer	Primary target
Nucleotide synthesis		
Actinomycin D	*Streptomyces* spp.	DNA
Bleomycin	*Streptomyces verticillus*	DNA, RNA
Calicheamicin	*Micromonospora echinospora*	DNA
Doxorubicin	*Streptomyces peucetius*	DNA
Mitomycin	*Streptomyces* spp.	DNA
Sterols		
Amphotericin B	*Streptomyces nodosus*	Ergosterol
Candicidin	*Streptomyces griseus*	Ergosterol
Natamycin	*Streptomyces natalensis*	Ergosterol
Nystatin	*Streptomyces noursei*	Ergosterol
Immunosuppression		
Ascomycin	*Streptomyces hygroscopicus*	FKBP12, Calcineurin
FK506	*Streptomyces tsukubaensis*	FKBP12, Calcineurin
Rapamycin	*Streptomyces hygroscopicus*	FKBP12, mTOR
Mitochondrial function		
Antimycin A	*Streptomyces* spp.	Cytochrome C reductase
Oligomycin	*Streptomyces distatochromogenes*	ATP synthase
Protein degradation		
Epoxomicin	*Streptomyces hygroscopicus*	20S proteasome
Salinosporamide A	*Salinospora* spp.	20S proteasome
Neurotransmission		
Avermectin	*Streptomyces avermectinius*	GluCl channel
Milbemycin	*Streptomyces hygroscopicus*	GluCl channel
Spinosyn	*Saccharopolyspora spinosa*	nACh receptor
Membrane		
Ionomycin	*Streptomyces conglobatus*	Lipid bilayer
Nigiricin	*Streptomyces hygroscopicus*	Lipid bilayer
Valinomycin	*Streptomyces* spp.	Lipid bilayer
Vacuolar pH		
Bafilomycin	*Streptomyces griseus*	V-ATPase
Concanamycin	*Streptomyces neyagawaensis*	V-ATPase
Signaling		
Lavendustin A	*Streptomyces griseolavendus*	Tyrosine kinase
Sangivamycin	*Streptomyces rimosus*	Protein kinase C
Staurosporine	*Streptomyces staurosporeus*	Protein kinase C
Other		
Borrelidin	*Streptomyces parvulus*	Threonyl-tRNA synthetase
Cycloheximide	*Streptomyces griseus*	60S ribosome
Geldanamycin	*Streptomyces hygroscopicus*	Hsp90
Leptomycin B	*Streptomyces* spp.	CRM1 (exportin)
Rebeccamycin	*Streptomyces* spp.	Topoisomerase I
Trichostatin A	*Streptomyces* spp.	HDAC (class I and II)
Tunicamycin	*Streptomyces* spp.	UDP-HexNAc

## Targeting DNA synthesis: doxorubicin

One of the mainstays of cancer chemotherapy involves the use of the anthracycline drugs epirubicin, pirarubicin, aclarubicin and idarubicin, all of which are derived from the foundational drug doxorubicin. These drugs are routinely used against malignancies such as adult acute leukemia, breast carcinoma, non-Hodgkin’s lymphoma and ovarian carcinoma [39, 89]. Indeed, the first clinically approved nano-drug (Doxil^®^) was a liposomally encapsulated form of doxorubicin used for the treatment of AIDS-induced Kaposi’s sarcoma and solid tumours [17].

**Fig. 1:**
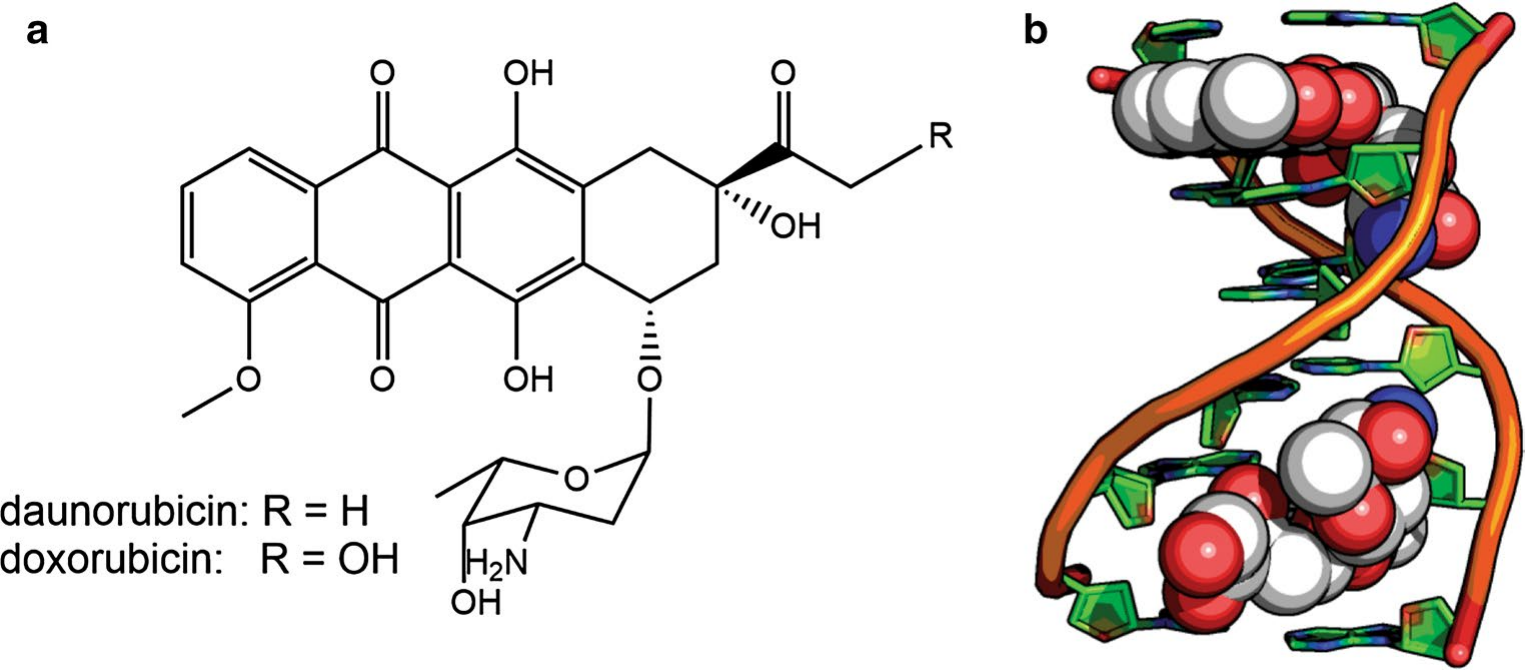
**a** The chemical structure of daunorubicin and doxorubicin (Prod. *S. peucetius*). **b** Crystal structure of DNA-doxorubicin associated complexes. The tetracyclic rings intercalate between base pairs while the aminosugar rests in the minor groove of DNA (PDB:1D12)

The first member of this class, daunorubicin, was isolated from *Streptomyces peucetius* in 1963 and found to be effective against murine tumours [48]. However, clinical trials revealed severe cardiotoxicity so the compound was abandoned [170]. In an effort to find a more therapeutically favourable analogue, Arcamone et al. mutagenized *S. peucetius* and isolated strains that produced an altered, and more clinically favourable form of the drug that was named doxorubicin [4]. Doxorubicin is still toxic however it can be dosed so as to maximize its anticancer activity and minimize damage to normal tissue. Both compounds are planar tetracyclic structures attached to an amino sugar moiety: doxorubicin differs from daunorubicin by a single hydroxyl group (Fig. 1a).

The earliest indication of doxorubicin’s mechanism of action came from in vivo assays showing reduced RNA synthesis in HeLa cells [49]. In that same year, Calendi et al. observed distinct changes in the physical properties of DNA when incubated with the drug in vitro [33]. Indeed, crystal structures [63] and NMR spectroscopy [195] of doxorubicin-DNA complexes show that the drug intercalates between the nitrogenous base pairs by planar insertion (Fig. 1b).

Doxorubicin was found to induce double strand breaks in the DNA of leukemic cells where the ends of the broken strands were associated with a protein complex. The protein was subsequently identified as topoisomerase II, the homodimeric enzyme responsible for relieving positive supercoiling by a double-strand cleavage and rejoining mechanism [172]. This and other work led to a model where doxorubicin intercalates DNA causing topoisomerase II to become trapped resulting in a ternary complex and a double-strand break [119]. The exact molecular mechanism of this process is not fully understood, however several mutagenesis studies in yeast implicate the CAP-like DNA-binding domain of topoisomerase II as a direct target [130, 144].

This model is widely recognized as doxorubicin’s primary mechanism of targeting proliferative cancerous cells in vivo. However there is support for alternative mechanisms in the literature. This includes most notably the generation of reactive oxygen species (ROS) [101] and gene-specific damage [35, 93]. It is possible that these alternative mechanisms occur simultaneously and are concentration dependant [66].

## Targeting fungal membranes: amphotericin B

Many antibiotics produced by actinomycetes target fungal cells [20]. The most clinically relevant class in this category are the polyene macrolides, particularly amphotericin B, isolated in 1953 at the Squibb Institute from the fermentation of *S. nodosus* [52]. Amphotericin B is a mainstay for managing systemic fungal infections. Amphotericin B is active against many fungal pathogen species in vitro including *Candida albicans* [10, 145]*, Aspergillus fumigatus* [8, 55]*, Cryptococcus neoformans* [10, 44]*, Blastomyces dermatitidis* [114, 166]*, Histoplasma capsulatum* [114]*, Rhizopus sp.* [55, 56] and *Mucroales sp.* [156]. Despite amphotericin B’s long-standing monotherapeutic use over the last 50 years, few resistant strains have emerged.

This is due to a tradeoff between tolerability and the fitness that is believed to limit resistance from developing [179]. However, a number of amphotericin B-resistant strains of *Aspergillus* [167], *Cryptococcus* [123] and *Candida* [190] have emerged in the clinic in recent years. In addition, amphotericin B treatment is often associated with adverse effects including nephrotoxicity [186] and anemia [116, 121, 193]. Interestingly, amphotericin B-induced anemia has been shown to occur through the inhibition of the transcription factor hypoxia-inducible factor-1 (HIF-1), thereby reducing the expression of erythropoietin (EPO) which controls red blood cell proliferation [121, 193].

**Fig. 2:**
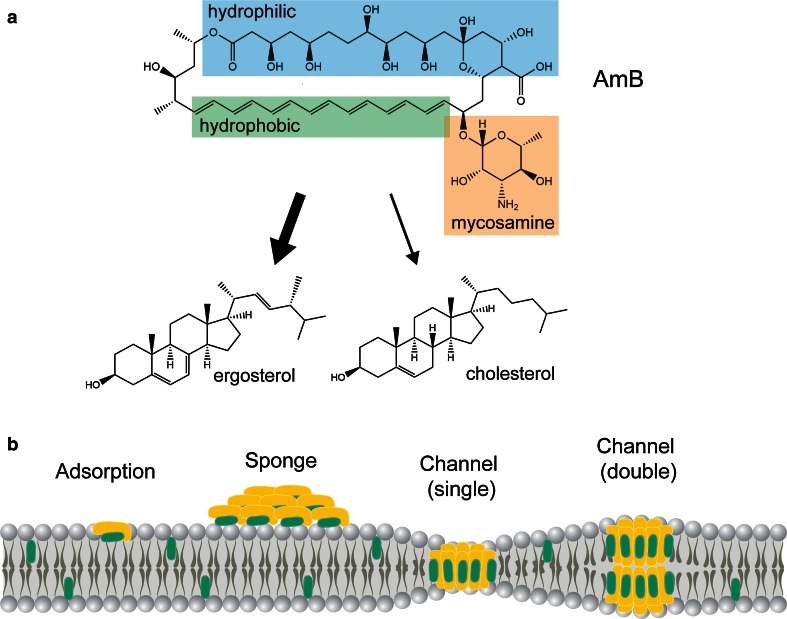
**a** The chemical structure of amphotericin B (Prod. *S. nodosus*). Highlighted are the drug’s molecular features that confer its specificity for ergosterol in fungi rather than cholesterol in mammalian cells. **b** Models of amphotericin B-ergosterol interactions with the lipid bilayer in fungi

Amphotericin B is comprised of an amphipathic macrolactone ring with a mycosamine attachment (Fig. 2a). These molecular features work together to bind fungal-specific sterols such as ergosterol in the membrane, causing ion-leakage [11, 28]. Hydrogen bonds formed between the mycosamine of amphotericin B and the hydroxyl group present in both ergosterol (fungal) and cholesterol (human) are essential for binding to occur [141]. Amphotericin B’s selective toxicity towards fungi is due to a more stable interaction between its seven conjugated double bonds with ergosterol [150, 177], presumed to be the result of reduced conformational flexibility [12].

An early model of amphotericin B’s mechanism of action was the ‘barrel-stave’ [125, 176]. In this model, eight amphotericin B-sterol complexes are aligned perpendicularly to the lipid bilayer forming a channel with the hydrophobic face on the exterior and the hydrophilic face pointing towards the interior (Fig. 2b). K^+^ ions would then leak out of the cell resulting in membrane depolarization and eventually cell death [5, 108]. Furthermore, amphotericin B forms ion channels more easily in the presence of ergosterol [88]. Recent studies by Gray et al. have challenged the notion that ion leakage by pore formation is the sole biochemical feature in its mechanism of action [70]. In particular, they found that a chemically modified analogue, C35deOAmB, lacking the ability to form pores, retained its antifungal potency. Likewise, the related polyene natamycin possesses antifungal activity despite its inability to form pores in the membrane [184]. An alternative model therefore, is that amphotericin B binds to the membrane monomerically, parallel to the lipid moieties (adsorption) [46, 131] or in aggregates (sponge) [2] to sequester ergosterol thereby causing a global reduction of sterol levels in the membrane. This in turn could limit the sterol’s function in maintaining the structural integrity and fluidity of the lipid bilayer as well as enabling the function of membrane-bound enzymes that influence a wide range of diverse signalling cascades [115, 185]. While it is likely that monomeric, aggregated and pore-forming states of amphotericin B occur simultaneously, the ratios at which these formations exist at various concentrations remain unknown.

Ongoing debate about its mechanism of action and toxicity suggests that modification of this drug, or the isolation and investigation of new congeners from other actinomycetes could drive the development of better antifungal drugs. More recently, novel derivatization of amphotericin B using diphenylphosphoryl azide (DPPA) led to two analogues: AmBMU and AmBAU, which were shown to be effective in evading resistance to *Candida* while having greater selectivity for ergosterol and were thus less toxic to human blood cells [45]. Notably, this study also revealed that amphotericin B-resistant strains of *Candida* are non-pathogenic in mice suggesting that minor changes in ergosterol significantly reduces pathogenicity. In addition, robust methods of synthesizing less toxic analogues of amphotericin B have been developed using the iterative cross-coupling of polyene building blocks which could potentially provide more potential candidates for improving drug efficacy [113].

It is widely agreed that additional antifungal drugs are needed to combat resistant strains and improve therapeutic outcomes associated with opportunistic mycoses including candiasis, cryptococcal meningitis and aspergillosis which often do not respond well to a limited number of current drug regimens [54, 146]. Indeed, fungal infections that were previously treated successfully with this drug are showing increasing resistance [67].

## Targeting cell growth (mTOR): rapamycin

The macrocylic lactone antibiotic rapamycin has had an enormous impact on medicine and on our understanding of eukaryotic cells. Its story began in 1964 when a Canadian expedition team collected soil samples from Easter Island in the southeastern point of the Polynesian Triangle in the Pacific Ocean. This soil sample was then investigated at Ayerst Laboratories in Montreal where the molecule, rapamycin (from Rapa Nui, the indigenous name for Easter Island), later derived from the isolated strain *S. hygroscopicus,* showed remarkable antifungal activity against *Candida* [178]. Persistent efforts led to rapamycin’s rise to acclaim where it was found to possess potent immunosuppressive and antiproliferative properties [86, 124], leading in turn to further investigation of its mode of action.

**Fig. 3:**
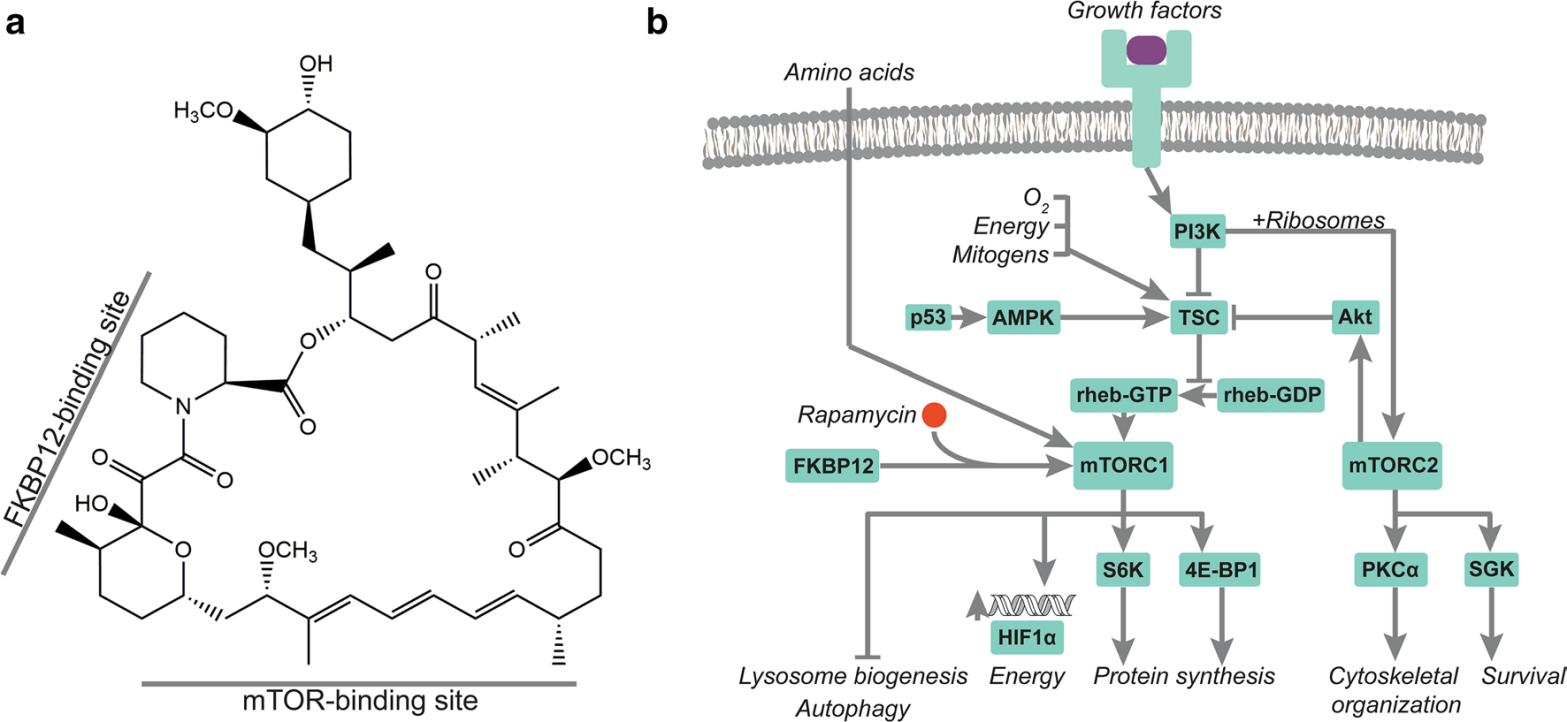
**a** The chemical structure and binding regions of rapamycin (Prod. *S. hygroscopicus*). **b** In mammals, rapamycin forms a ternary complex with FKBP12 (FK506-binding protein 12) and mTOR within the mTORC1 (mammalian target of rapamycin complex 1). This pathway is implicated in sensing environmental cues that regulate major cellular outputs

Initially, the structurally related immunosuppressant FK506 was found to target the 12-kDa FK506-binding protein (FKBP12), a peptidylprolyl romatase [81, 164]. The complex then acquires a gain-of-function ability to suppress the activation of T-cells in the immune system through a third target, calcineurin [104]. Similarly, rapamycin also binds to FKBP12 however mounting evidence suggested that FK506 and rapamycin varied in their mechanism of immunosuppression in murine T-cells [23, 50, 51], suggesting that the tertiary target of the rapamycin-FKBP12 complex was not the same as FK506. A landmark study by Heitman et al. was carried out in the budding yeast *Saccharomyces cerevisiae* in which genetic screens led to the identification of dominant mutations in *TOR1* and *TOR2* that were shown to confer rapamycin resistance [83]. This suggested that the encoded TOR (target of rapamycin) proteins—paralogous serine/threonine kinase subunits—were the targets of the FKBP-rapamycin complex that ultimately resulted in immunosuppression and growth reduction. It was subsequently found that rapamycin binds proteins in a mammalian cells that shared extensive sequence similarity to the TOR1, providing not only direct evidence of the rapmaycin-FKBP binding targets but also showing that the mechanistic targets are highly conserved in lower and higher eukaryotes [29, 42, 153, 154]. X-ray crystallography further elucidated the drug’s mode of action showing that rapamycin has two binding sites [13, 37] (Fig. 3a). Most eukaryotic organisms possess one TOR protein. The mTOR (mammalian target of rapamycin) is a large (289 kDa) protein that belongs to the phosphoinositide kinase-related kinase (PIKK) family. It associates with other proteins to form two functionally distinct complexes: mTORC1 and mTORC2.

Proteins that act upstream of mTORC1 mediate intracellular responses to a variety of intra- and extracellular cues: growth factors [61], oxygen levels [9], energy [27, 102], mitogens [38, 57] and amino acids [10, 25]. TSC 1/2 (tuberous sclerosis 1 and 2) are key upstream regulators that inhibit mTORC1 by repressing the formation of the GTP-bound state of Rheb (Ras homolog enriched in brain) [91, 171]. Two downstream effectors phosphorylated by mTORC1 are the eukaryotic translation initiation factor 4E (eIF4E)-bindng protein 1 (4E-BP1) and S6 kinase (S6K) [21, 30, 32, 80]. The current model posits that mTORC1 senses environmental cues and works to positively regulate downstream signals of protein synthesis by controlling components within the translation machinery (Fig. 3b).

The mTORC2 signalling network was initially thought to be rapamycin insensitive [95, 157] however, recent studies suggest that mTORC2 does respond to rapamycin in certain cell types after prolonged exposure to the drug [147, 158]. Less is known about the mTORC2 pathway however it has been shown to associate with the ribosome and is required for activation [196]. mTORC2 has been shown to regulate three kinases: Akt [159], serum- and glucocorticoid-induced protein kinase 1 (SKG1) [65] and protein kinase C-α (PKCα) [157]. Akt works to phosphorylate downstream processes of survival, apoptosis, growth and proliferation as well as directly feedback into mTORC1 signaling through the inhibition of TSC [72, 92, 94], SKG1 affects ion transport and growth [162] and PKCα affects the remodelling of the actin cytoskeleton [87, 95, 157].

In addition to mediating rapamycin’s clinical use for preventing graft rejection after organ transplantation and for treating autoimmune disorders, the components of the mTOR pathway have also been implicated in many other conditions including obesity related type 2 diabetes [105, 112, 148, 174, 175] and cancer [73, 182, 189]. Indeed, mutations in negative and positive regulators of mTOR signaling are among the most common tumour suppressors and oncogenes that arise in cancer patients. As a result, a number of rapamycin derivatives (rapalogues) have been approved for the treatment of various cancers [18]. More recently, rapamycin has been explored as a treatment for age related diseases after the drug remarkably was shown to increase the lifespan of yeast [149], nematodes [152], fruit flies [24] and mice [3, 82, 129].

Rapamycin exemplifies the enormous value that eukaryotic targeting compounds can have through the exploration of the drug’s mode of action. In addition to the drug and its derivatives being useful therapeutics with a variety of applications, they serve as chemical probes that can be used to elucidate the inner workings of complex biological pathways.

## Targeting neurotransmission: avermectin

**Fig. 4:**
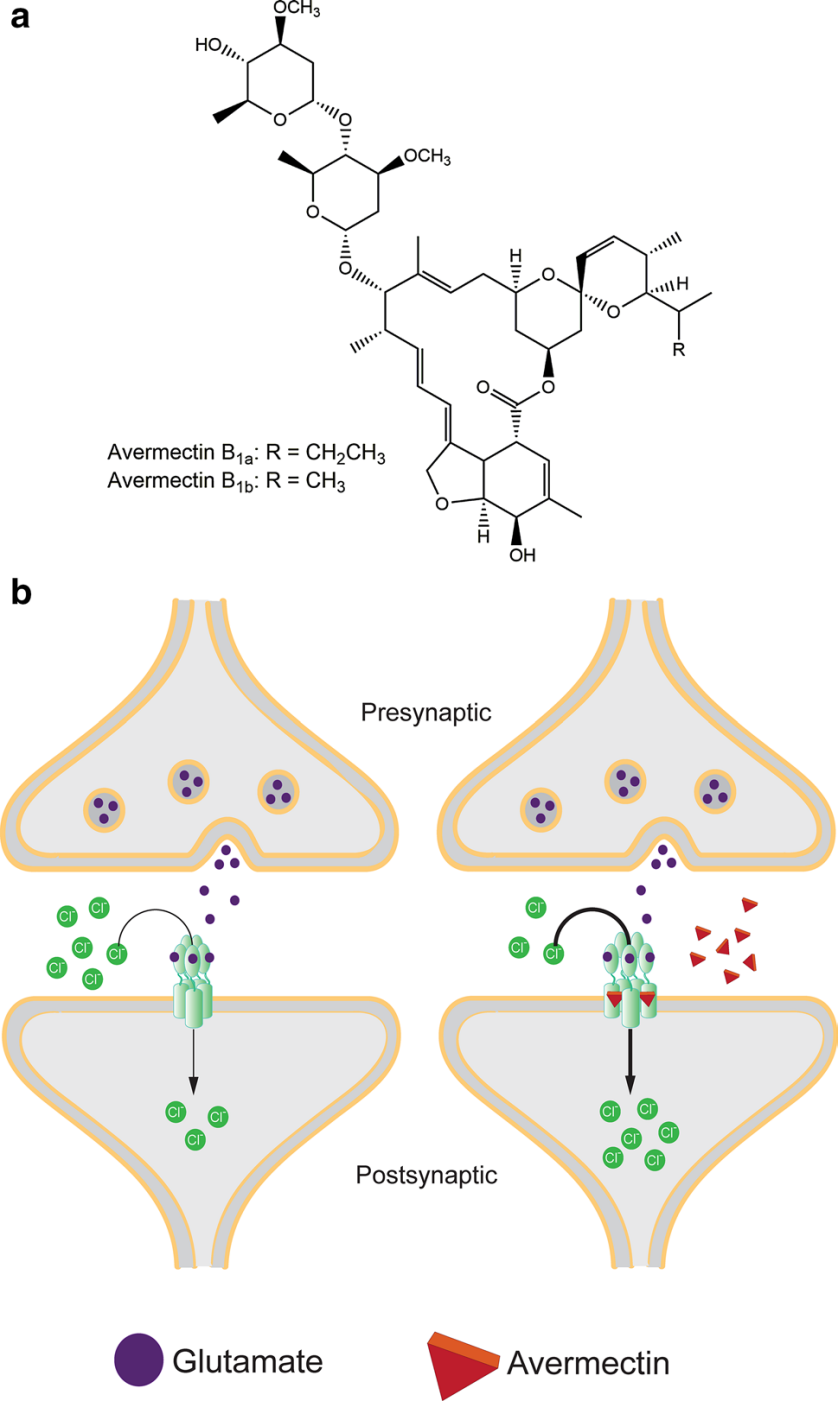
**a** The chemical structure of avmermectin B1a and B1b (Produced by *S. avermitilis)*. **b** (*Left*) At the neuromuscular junction, action potentials are transmitted from the presynaptic to the postsynaptic cell via the neurotransmitter glutamate binding to the GluCl (glutamate-gated chloride channel) allowing the influx of chloride ions. (*Right*) Avermectin acts as a GluCl agonist by increasing ion influx resulting in irreversible hyperpolarization

The avermectins are a class of macrocyclic lactones that have broad-spectrum activity against nematodes and insects, but that lack antimicrobial activity. In the 1970s researchers at the Kitasato Institute isolated *S. avermitilis* (also referred to as *S. avermectinius*) from a soil sample on a golf course in Shizuoka Prefecture, Japan. The fermentation of this microbe was found to have potent activity against helminth parasitic worm *Nematospiroides dubius* and remarkably cured the worm-infected mice with little to no toxicity [31]. Soon following, these compounds were identified as a mixture of eight isomers of which avermectins B_1a_ and B_1b_ were found to be the most potent derivatives [139] (Fig. 4a).

Avermectin disrupts glutamate-gated chloride channels (GluCls) in nematodes [6, 7] and insects [43, 99] that play a critical role in muscle contraction required for locomotion and feeding. The GluCl channel belongs to a Cys-loop receptor family and is comprised of five subunits. These respond to glutamate to allow the influx of chloride ions to transmit an action potential from the presynaptic to the postsynaptic neuron (Fig. 4b). Avermectin disrupts this process by irreversibly inserting itself between the transmembrane domains thereby causing ions to constitutively leak through the compromised channel [84]. This results in the hyperpolarization of the neuromuscular synapses causing paralysis and subsequent death. It is selective for nematode parasites because mammals do not have GluCls but instead have the evolutionarily related gamma-aminobutyric acid (GABA) receptor channels [188]. While avermectin can bind GABA receptors in the mammalian central nervous system, the pharmacological effectiveness of the drug is owed to its inability to cross the blood–brain barrier [160, 161].

Initially, avermectin was studied for use in veterinary medicine and animal husbandry. The medical formulation of the drug, ivermectin, became useful in agriculture, saving livestock affected by ectoparasitic arthropods and endoparasitic helminth nematodes [34]. But the most significant contribution that this drug has had was its use to treat river blindness, a disease caused by the parasite *Oncocerca volvulus* that is transmitted by the black fly. Ivermectin is credited for significantly reducing morbidity and transmission of onchocercal infections in the endemic regions of sub-Saharan Africa and Latin America preventing an estimated 600,000 cases of river blindness [26].

## Targeting nucleo-cytoplasmic transport: leptomycin B

Like many compounds that target the eukaryotic cell, leptomycin B and its derivatives were originally identified in screens for antifungal and antitumor antibiotics [77–79, 110]. In 1994, Nishi et al. identified a mutant of the *crm1* (chromosome region maintenance) gene that conferred leptomycin B resistance in fission yeast [135]. This gene, previously reported by Adachi and Yanagida [1], affected higher order chromosomal structure and resulted in an identical phenotype when mutated compared to leptomycin B-treated cells [135]. This provided strong evidence that the molecular target of leptomycin B was CRM1, a protein that belongs to the importin-β-like family of nuclear transport machinery that mediates the export of proteins and RNAs out of the nucleus [192].

In order to understand how leptomycin B works, it is important to first recognize the role that the nuclear envelope plays in the cell. That is, the physical separation of the genome and cytoplasm, a central feature of eukaryotic cells. The trafficking of proteins and RNA is a highly coordinated process that takes place across the nuclear envelope which is contiguous with the endoplasmic reticulum and contains anywhere between 200 and 2000 nuclear pore complexes (NPC) that facilitate bi-directional transport between the nuclear and cytoplasmic compartments.

**Fig. 5:**
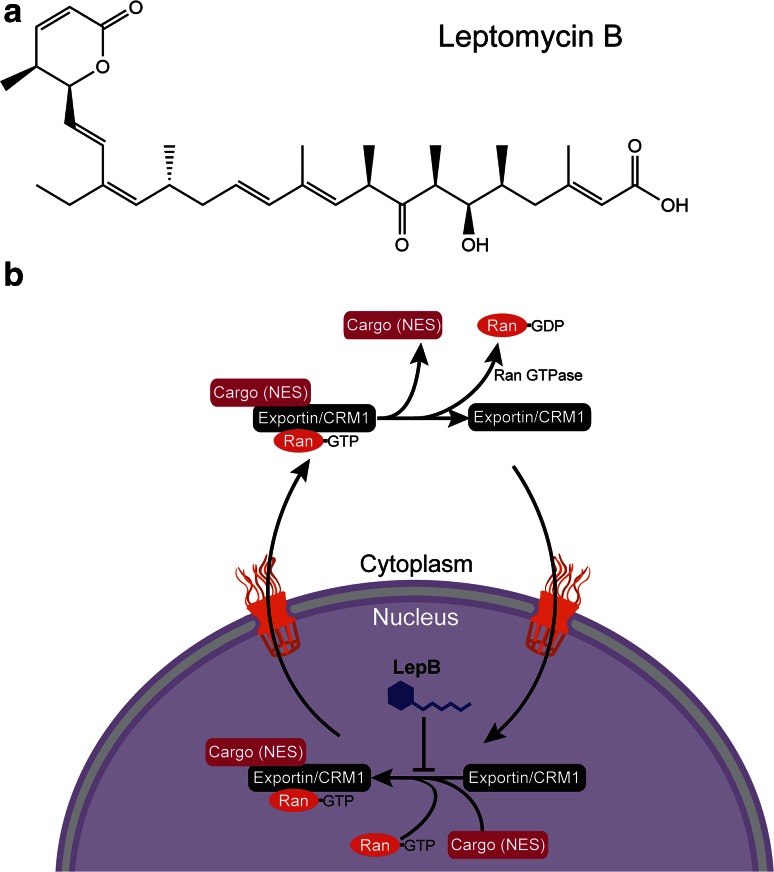
**a** The chemical structure of leptomycin B. **b** Nucleo-cytoplasmic transport of protein cargo with a leucine-rich NES (nuclear export signal) by exportin/CRM1. Leptomycin B inhibits the loading of exportin with the cargo and Ran-GTP by alkylation

Later, in a screen carried out by Wolff et al. leptomycin B was identified as an inhibitor of the nuclear export of Rev, a protein required for trafficking of HIV-1 mRNA from the nucleus to the cytoplasm [187]. This coincided well with the fact that leptomycin B prevents the cargo-loading of proteins that carry leucine-rich nuclear export signals (NES) that are to be transported to the cytoplasm through the nuclear pore [62] (Fig. 5b). It does so by forming a covalent bond with CRM1 where inactivation is thought to occur by a Michael-type addition between the α, β- unsaturated lactone terminus of the compound and a key cysteine residue that is essential for leptomycin B sensitivity [111] (Fig. 5a).

The specificity of leptomycin B has been used to validate the CRM1-dependent export of many NES- containing proteins including actin [181], cytokines [140], tyrosine kinases [169], cyclin-CDK [75, 191], MDM2/p53 [64], inhibitors of NF-κB transcription [155] and MHC class II complexes [36]. Inhibition by this drug results in the accumulation of these key regulatory proteins which eventually leads to cell death.

Efforts have been made to improve the therapeutic efficacy through the synthesis of leptomycin B semi-synthetic derivatives [132]. However, in contrast to many of the well-known actinomycete-derived molecules that target eukaryotic organisms, leptomycin B has gained most of its notoriety as a powerful experimental tool to probe biological complexity.

## Targeting the proteasome: epoxomicin

The 20S proteasome is found in all eukaryotic cells where it serves to degrade proteins during their natural turn-over cycle or proteins that have been misfolded or have sustained other damage. One way that proteins are targeted for proteolysis is via a post-translational modification called ‘ubiquitination’. This involves the ligation of a small regulatory protein called ubiquitin to the protein; ubiquitin is then recognized by the proteasome resulting in targeting of the modified adduct for degradation [68].

The *α’,β’*-epoxyketone epoxomicin specifically targets proteasomes, a key protease of intracellular protein degradation. Epoxomicin was discovered in 1992 through a screening programme at Bristol-Myers Squibb in Tokyo, Japan and is produced by the unidentified actinomycete strain Q996-17 where it was initially reported having antitumor activity against B16 melanoma cells in mice [76].

**Fig. 6:**
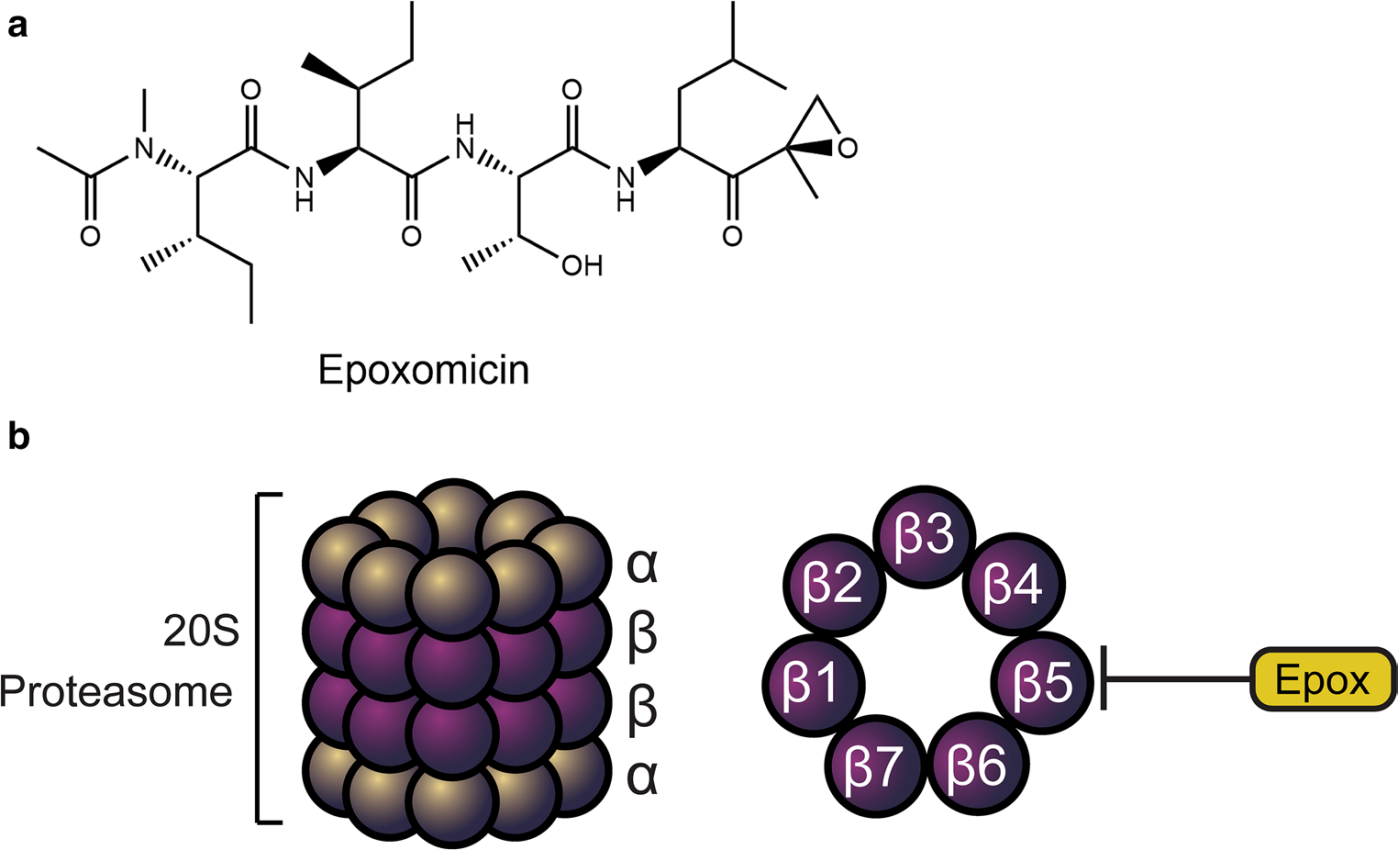
**a** The chemical structure of epoxomicin. **b** Epoxomicin inhibits the β5 subunit of the core 20S proteasome thereby inhibiting protein degradation

The chemical structure of epoxomicin consists of four linked peptides with an unusual terminal epoxy ketone group (Fig. 6a). This chemical moiety is highly reactive and is therefore considered the ‘warhead’ or ‘pharmacophore’ of the drug due to the triangular epoxy ring having highly strained 60° bond angles which are more stable once decyclized by nucleophilic attack. This inherent instability led to the near abandonment of further development of the drug [100]. However, efforts to understand the epoxomicin’s mode of action were continued hoping to gain a better understanding of its antitumor activity.

The peptidic nature of epoxomicin allowed Meng et al. to synthesize the drug with ease which was then biotinylated to chemically attach and immobilize the drug to an affinity column [128]. This, remarkably, led to the epoxomicin-binding proteins being identified as components of the catalytic β subunits of the 20S proteasome: low-molecular mass polypeptide-7 (LMP7, β5i), subunit X (PSMB5), which confer chymotrypsin-like activity and multicatalytic endopeptidase complex like 1 (MECL1, β2i), subunit Z (β2), which confer trypsin-like activity to the proteasome. Consistent with the fact that epoxomicin preferentially inhibits the β5 subunit of the core proteasomal particle, epoxomicin is highly selective against chymotrypsin-like activity—that is the inhibition of protein cleavage after aromatic and hydrophobic amino acid residues such as tyrosine, tryptophan and phenylalanine [53, 128] (Fig. 6b).

Groll et al. then co-crystallized epoxomicin bound to the yeast 20S proteasome to elucidate the exact molecular mechanism of the epoxomicin-proteasome interaction. The three-dimensional molecular interaction between the drug and the proteasomal catalytic subunit revealed that a covalent linkage with the N-terminal threonine of the proteasome forms a six-membered morpholino ring [71, 183]. Also showing that epoxomicin fits well into the pocket surrounding the threonine residue within the active site, preferentially binding to the chymotrypsin-like pocket, and at higher concentration than the trypsin-like pocket.

To understand epoxomicin’s activity on a cellular level, we will shortly recapitulate the function of the proteasome, the key protease for short-lived proteins regulating a broad variety of cellular processes such as cell cycle progression, gene expression, protein quality control and stress response. Well known proteasomal substrates include cyclins [14, 22], caspases [133, 168], p53 [122], p27 [120] BCL2 [118] and nuclear factor κB (NF- κB) [142]. The inhibition of their proteolysis triggers apoptosis. Thus, chemically induced apoptosis by proteasome inhibitors such as Bortezomib (Velcade^®^) are successfully used to combat the progression of certain cancer cells.

Several lines of evidence suggest a heightened dependency on protein quality-control mechanisms mediated by the ubiquitin–proteasome system in cancer cells [74, 85, 120]. Because of this, epoxomicin in combination with proteasome inhibitors are exceptional candidates as antineoplastic therapeutics that can have a very potent cytotoxic effects in cancer cells. In phase I and II clinical trials, inhibition of the 20S proteasome is highly cytotoxic to plasma cell cancer multiple myeloma [163] and mantle cell lymphoma [137]. The high expression of proteasomes in proliferative blood cells also suggests that proteasome inhibitors are potentially suited to haematopoietic malignancies [97]. The drug form of epoxomicin (Carfilzomib) is now released as an FDA-approved treatment for relapsed multiple myeloma and is currently undergoing phase III clinical trials [96, 100, 126, 180]. Presumably, higher expression of proteasomes in blood cells compared to peripheral tissues may diminish the drug’s access to solid tumors which may limit proteasome inhibitors to blood cancers [47].

Epoxomicin is a rare compound that specifically targets a unique process in eukaryotic organisms, namely chymotrypsin-like activity of the proteasome. Similarly, some other actinomycete-derived proteasome inhibitors: lactacystin [58] and salinosporamide [59], also inhibit the β5 catalytic subunit of the 20S proteasome which suggests that the proteasome may be a common target for natural products of microbial-origin. There are likely a number of natural products that inhibit the proteasome yet to be discovered.

## Eukaryotic targets are more diverse than prokaryotic targets

### Target diversity

The targets of antibacterials are conspicuously concentrated in four pathways: DNA synthesis, RNA synthesis, protein synthesis and cell wall synthesis [106]. Indeed, there are often multiple targetable proteins in each pathway. For example, tetracycline targets the small 30S ribosomal subunit, chloramphenicol targets the large ribosomal subunit and kirromycin targets EF-Tu. Aside from a few minor antibiotics and antibiotic targets (e.g., platensimycin inhibits fatty acid biosynthesis and daptomycin disrupts the cell membrane) these central components of macromolecular synthesis are the targets of virtually all naturally occurring antibiotics that are known at this time.

**Fig. 7:**
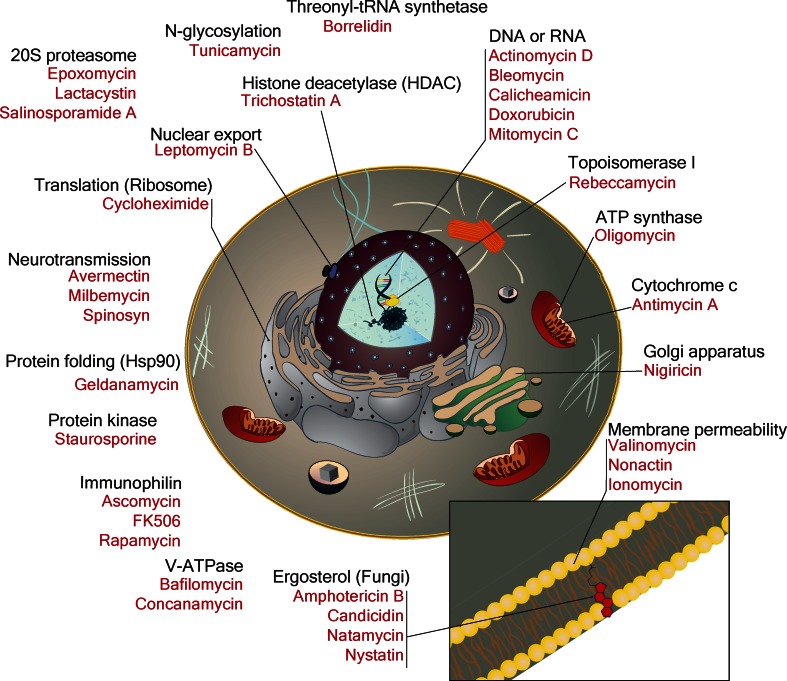
The highly diverse eukaryotic targets of actinomycete metabolites

To date we have identified far fewer eukaryote-active compounds than prokaryote-active compounds among the secondary metabolites produced by actinobacteria. However, the contrast in target diversity could not be greater (Fig. 7). Indeed, Table 1 reveals at least 20 distinct molecular targets in most of the major organelles of the eukaryotic cell. In fact, the common antibacterial targets (DNA synthesis, RNA synthesis and cell wall synthesis) are under-represented relative to the high number of other identified molecular targets in eukaryotic cells.

There are many biosynthetic processes for which there are no natural product inhibitor, and others for which there are only one or two that are known. This, again, is in marked contrast with the antibiotics where dozens of distinct compounds are known that inhibit common targets within bacteria. This suggests that we have yet to reach saturation of possible eukaryotic targets and that additional compounds of interest await discovery. We wonder in particular, whether there are bacterially produced inhibitors of peroxisome biogenesis and function, centrosomes, or even components of key signaling pathways like the Janus kinase/signal transducers (JAK-STAT) pathway, the mitogen-activated protein kinase (MAPK) cascade or regulators of the cell cycle cascade.

How might these questions be addressed? We suggest, as a foundational concept, that any eukaryotic pathway that has been in contact with actinobacteria since its appearance in evolutionary time has a potential biochemical target for a secondary metabolite. The remarkable (though far from exhaustive) description of compound/target interactions that we have provided certainly supports this. Therefore, we propose that more concerted screening campaigns of actinobacterial metabolites against model eukaryotes is a timely and exciting response to this question. These screens should harness more than just live/dead screening. In other words, we should look for interesting developmental and behavioural phenotypes using the well-developed model systems. And in designing screens, we should harness the known molecular biology of the various pathways of interest. In doing so, and by avoiding simple live/dead screens only against microbes, we could avoid the rediscovery of known compounds such as daunorubicin, bafilomycin and cycloheximide and focus our attention on novel chemical scaffolds.

For example, the roundworm *Caenorhabditis elegans* could be screened for compounds that act via components the nervous system and confer motility defects [98]. The fly *Drosophila melanogaster* could be screened for compounds that interfere with a myriad of developmental pathways, most of which are conserved in humans [143]. Fruiting body formation in the amoeboid organism *Dictyostelium discoideum* could serve as a reporter for cell adhesion and cell sorting [60]. The simple mustard plant *Arabidopsis thaliana* offers numerous possibilities for the identification of compounds that interact with the photosynthetic apparatus or other conserved pathways in the plant Kingdom [107]. Indeed, chemical perturbation of the life cycles of many well-characterized eukaryotic organisms also offers the potential for unique insights into both morphogenesis and hidden mechanistic details of eukaryotic cell biology.

We note that the road blocks encountered in antibacterial screening will also be encountered in these searches. Low levels of expression of many secondary metabolic compounds would necessitate strategies for the search for cryptic metabolites, many of which now exist. And the rediscovery of known compounds will also confound some screens. However, this confluence of technologies, the rapidly expanding database of actinobacterial genomes, and the wide-spread interest in chemical inhibitors of eukaryotic life suggests that the time has never been better for a concerted search for new eukaryote-active secondary metabolites.
